# Mining the oil code: New insights behind oil production in *Brassica napus*

**DOI:** 10.1093/plphys/kiae457

**Published:** 2024-08-28

**Authors:** Sebastián R Moreno

**Affiliations:** Assistant Features Editor, Plant Physiology, American Society of Plant Biologists; Sainsbury Laboratory, University of Cambridge, Cambridge CB2 1LR, UK

*Brassica napus*, commonly known as rapeseed, ranks as the third-largest source of vegetable oil in the world, contributing approximately 13% to the world's edible oil (USDA ERS 2020). Plant oils are stored mainly as triacylglycerols during seed development, serving as a major energy reserve to support seed germination and seedling development (Yang et al. 2022a). With global demand for vegetable oil rapidly increasing and projected to double by 2030 (Chapman and Ohlrogge 2012), enhancing seed oil content (SOC) has emerged as a crucial breeding objective for *B. napus*.

Seed oil production is orchestrated by an interplay of environmental and developmental cues that influence a network of numerous transcription factors (TFs). The LAFL network involving LEAFY COTYLEDON1 (LEC1), LEAFY COTYLEDON2 (LEC2), FUSCA3 (FUS3), and ABSCISIC ACID INSENTITIVE3 (ABI3) plays a central role in multiple aspects of seed development and oil biosynthesis (Yang et al. 2022a). Enhanced expression of the LAFL network has been associated with increased seed oil production across different species (Parcy et al. 1997; Yang et al. 2022b). Additionally, WRINKLED1 (WRI1), regulated by LEC1, FUS3, and ABI3 (Wang and Perry 2013; Pelletier et al. 2017; Tian et al. 2020), is also crucial for determining SOC, with *wri1-1* mutant lines exhibiting nearly 80% reduction in SOC compared with wild-type plants (Focks and Benning 1998).

Understanding the molecular mechanism underlying complex traits has been a major focus to improve crop performance. Approaches such as expression genome-wide association study (eGWAS) links genomic variations with transcriptomics datasets to elucidate the key molecular players affecting traits. Previous studies have identifies genetic variants using single-locus models in the seed transcriptome of *B. napus* (Tan et al. 2022), revealing the roles NAC13 and SCL31 impacting SOC-related genes. However, multi-locus models, which provide a more comprehensive analysis of gene regulatory network, have never been assessed to study seed oil production in *B. napus*.

In this issue of *Plant Physiology*, Han and colleagues aimed to understand the genetic determinates controlling oil production in *B. napus* during seed development. Utilizing eGWAS with multi-locus methods, the researchers examined 302 *B. napus* accession and 583 seed transcriptomes to identify specific DNA variations influencing the expression of SOC-related genes. The study focused on seeds at 20 and 40 days after flowering (DAF), a critical period when embryo growth commences and oil accumulation stabilizes, respectively (Fig.).

**Figure.**
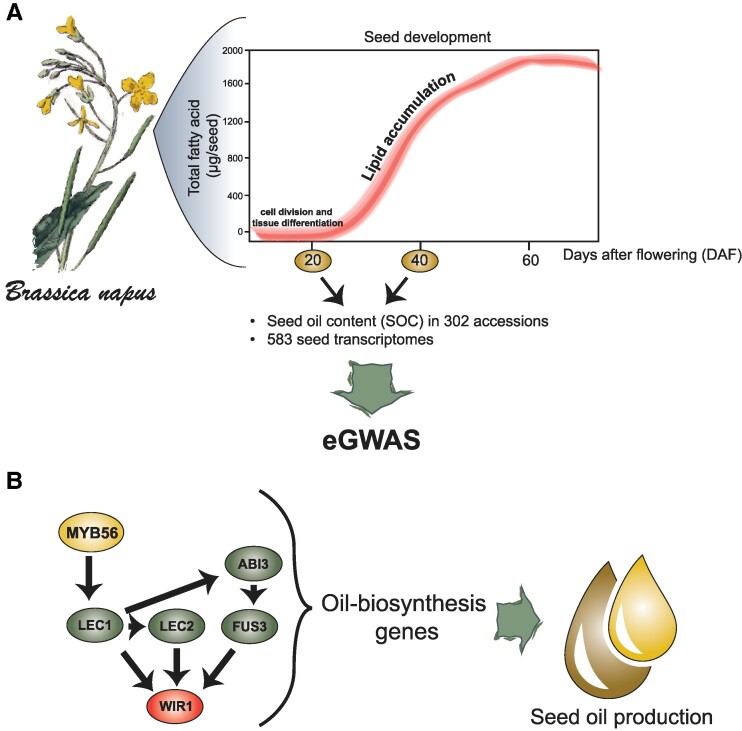
Mining genes through eGWAS uncovered the role of MYB56 in seed oil production. **A)** eGWAS analysis were performed in *Brassica napus* seeds 20 and 40 DAF. **B)** Machine-learning approaches and coexpression regulatory networks uncovered MYB56 as a new gene in control of seed oil production in seeds.

The authors' analysis identified novel candidates with known transcriptional or protein interaction roles with respect to their expression traits. The identified genetic variants associated with gene expression that were significant for at least 2 genes were selected for subsequent analysis. Employing machine learning–based approaches trained on multi-tissue ATAC-seq datasets, the authors predicted regulatory factors within the promoter regions of WRI1 and LAFL genes (Kelley et al. 2018). Consequently, 10 known genes and 12 newly predicted genes were implicated as TFs regulating the expression of the *WRI1-LAFLs* network. The validation of these candidates involved the expression profiles at different tissues, haplotype analysis, and expression correlation analysis. Furthermore, coexpression networks analysis at 20 and 40 DAF revealed modules involving *WRI1-LAFLs* and the newly identified candidates such as *BnaAGL15*, *BnaMYB56*, and *BnaVAL1* (Fig.). Gene Ontology (GO) enrichment analysis supported the significance of this module in SOC, with terms related to seed maturation, lipid metabolism, and seed morphogenesis being differentially enriched.

Finally, to substantiate the regulatory role of MYB56, the researchers conducted transient dual luciferase and yeast 1-hybrid assays, confirming the direct binding of MYB56 to the LEC1 promoter. From the several newly TFs observed in the coexpression module, the authors focused on *BnaMYB56* because of its potential pleiotropic effect in seed development. To validate the role of MYB56 in oil production, the authors quantified SOC in wild type and *myb56 A. thaliana*, observing a reduction in SOC and downregulated expression levels of *LEC1*, *WRI1*, and various lipid-related biosynthesis genes. Concomitantly to the pleiotropic effect of MYB56 suggested by coexpression network analysis, the seed length and seed area of *myb56* mutants were significantly smaller compared with wild-type plants.

In conclusion, the findings provided by Han et al. offered profound new insights into the transcriptional regulation of seed development and oil production in *B. napus*, identifying MYB56 as a potential target for enhancing oil yield. Further exploration of SOC in *B. napus* upon MYB56 disruption could provide valuable avenues for crop improvement. Moreover, this study underscores the utility of multi-locus eGWAS analysis in unravelling the genetic architecture of agriculturally significant complex traits, such as seed oil production.
